# Towards the knowledge-based design of universal influenza epitope ensemble vaccines

**DOI:** 10.1093/bioinformatics/btw399

**Published:** 2016-07-10

**Authors:** Qamar M. Sheikh, Derek Gatherer, Pedro A Reche, Darren R. Flower

**Affiliations:** ^1^School of Life & Health Sciences, Aston University, Aston Triangle, Birmingham B4 7ET, UK; ^2^Division of Biomedical & Life Sciences, Faculty of Health & Medicine, Lancaster University, Lancaster LA1 4YW, UK; ^3^Facultad de Medicina, Departamento de Microbiologia I, Universidad Complutense de Madrid, Madrid, Spain

## Abstract

**Motivation:** Influenza A viral heterogeneity remains a significant threat due to unpredictable antigenic drift in seasonal influenza and antigenic shifts caused by the emergence of novel subtypes. Annual review of multivalent influenza vaccines targets strains of influenza A and B likely to be predominant in future influenza seasons. This does not induce broad, cross protective immunity against emergent subtypes. Better strategies are needed to prevent future pandemics. Cross-protection can be achieved by activating CD8+ and CD4+ T cells against highly conserved regions of the influenza genome. We combine available experimental data with informatics-based immunological predictions to help design vaccines potentially able to induce cross-protective T-cells against multiple influenza subtypes.

**Results:** To exemplify our approach we designed two epitope ensemble vaccines comprising highly conserved and experimentally verified immunogenic influenza A epitopes as putative non-seasonal influenza vaccines; one specifically targets the US population and the other is a universal vaccine. The USA-specific vaccine comprised 6 CD8+ T cell epitopes (GILGFVFTL, FMYSDFHFI, GMDPRMCSL, SVKEKDMTK, FYIQMCTEL, DTVNRTHQY) and 3 CD4+ epitopes (KGILGFVFTLTVPSE, EYIMKGVYINTALLN, ILGFVFTLTVPSERG). The universal vaccine comprised 8 CD8+ epitopes: (FMYSDFHFI, GILGFVFTL, ILRGSVAHK, FYIQMCTEL, ILKGKFQTA, YYLEKANKI, VSDGGPNLY, YSHGTGTGY) and the same 3 CD4+ epitopes. Our USA-specific vaccine has a population protection coverage (portion of the population potentially responsive to one or more component epitopes of the vaccine, PPC) of over 96 and 95% coverage of observed influenza subtypes. The universal vaccine has a PPC value of over 97 and 88% coverage of observed subtypes.

**Availability and Implementation:** http://imed.med.ucm.es/Tools/episopt.html.

**Contact:** d.r.flower@aston.ac.uk

## 1 Introduction

Vaccines are the most efficacious and efficient medical intervention known. While vaccines based on inactivated or attenuated whole microbes or single protein subunits have proved widely useful, vaccines based on ensembles of epitopes remain underexploited. We have recently developed a powerful new approach to the design of epitope ensemble vaccines combining available experimental data with informatics-based immunological predictions (Molero-Abraham *et al.*, 2013). This approach now begins to gain a gratifyingly widening acceptance as a vaccine design tool (Gededzha *et al.*, 2014; Oany *et al.*, 2015).

Influenza is an infectious acute respiratory disease caused by single-negative-strand RNA viruses of the family Orthomyxoviridae, within which three genera—*Influenzavirus A*, *Influenzavirus B* and *Influenzavirus C*—contain viral strains corresponding to influenza types A, B and C, respectively (ICTV, 2015; http://ictvonline.org/virusTaxonomy.asp). Treatment when necessary includes antivirals to relieve symptoms and reduce complications. However, prevention through vaccination is currently the most effective strategy to combat influenza viruses (Schotsaert and Garcia-Sastre, 2014).

Only influenza A has both seasonal epidemic and pandemic capability and is classified into subtypes according to its hemagglutinin (HA) and neuraminidase (NA) surface glycoprotein antigens (Zhang *et al.*, 2015). Influenza A caused the Spanish (1918–1919), Asian (1957) and Hong Kong (1968) flu pandemics, which all led to global mortality in the millions (Kilbourne, 2006), as well as the milder 2009 pandemic. Pandemic strains from both 2009 (H1N1) and 1968 (H3N2) continue to circulate as seasonal influenza maintained by antigenic drift where mutations occur on the antigenic binding sites of NA and HA. However, pandemic strains arise through antigenic shifts, where a new subtype emerges via gene re-assortment after distinct influenza viruses co-infect an intermediate host.

Seasonal flu vaccines are mediated by antibody responses against NA and HA proteins, which unfortunately are highly variable; the large numbers of annual deaths continues to raise serious questions about the effectiveness of current vaccines and vaccination strategies (World Health Organisation [WHO], 2009; http://www.who.int/csr/don/2009_09_18/en/). Formulating a ‘universal vaccine’ where multiple strains are targeted to induce so-called heterosubtypic immunity (HSI) may prove more effective in combating novel strains before they can go on to cause pandemics (Nguyen *et al.*, 2007).

Studies in animal models have suggested that generating cellular immunity via T cell responses may induce broad, cross-reactive protection that current vaccines apparently lack. T cells target exposed and non-exposed proteins that are processed and presented by Human Leukocyte Antigens (HLAs). There are two main types of T cells: cytotoxic CD8+ T cell lymphocytes (CTLs) and CD4+. CTLs recognize antigens presented by HLA class I (HLA I) molecules while CD4 T cell recognize antigens presented by HLA class II (HLA II) molecules. The role of CTL in cellular immunity includes the direct clearance of virally infected cells and the indirect recruitment of other immune cells via chemokine and cytokine secretion. CD4+ T cells' primary roles include B cell stimulation leading to specific antigen antibody production as well as stimulating CD8+ proliferation and memory responses. CD4+ T cells also mediate direct and indirect viral clearance, and symptom severity reduction in secondary infection (Chen *et al.*, 2014).

Many targets for influenza vaccine-development have been investigated, including those inducing T cell and/or B cell responses; e.g. the highly conserved ‘stalk’ domain of HA, termed HA2 (Khanna *et al.* 2014). Other evidence has implicated nucleoprotein (NP) and Matrix 1 (M1) as vaccine targets capable of inducing cross protection via enhanced T cell activation (Antrobus *et al.*, 2014a,b).

Another potential vaccine target is influenza matrix protein 2 (M2e). Initially this seemed highly conserved and induced broadly acting antibodies. However, over 20 variants of M2e were later identified in newly emerging influenza A strains, raising questions over its usefulness as a ‘universal’ vaccine. Nevertheless, regions of M2e may still be useful for inducing broadly acting protection (Gottlieb and Ben-Yedidia, 2014).

Conserved targets would be useful in formulating a ‘universal’ vaccine, as they would cover multiple viral subtypes (Brown and Kelso, 2009). Such ‘universal’ vaccine design can potentially be addressed by a T-cell epitope ensemble vaccine comprising short, highly conserved, immunogenic peptides from influenza able to activate T cells. Some epitope-based influenza vaccines are already in clinical trials. One, Multimeric-001, which consists of a single recombinant protein composed of B and T cell epitopes, is seen to induce both cellular and humoral immunity (Atsmon *et al.*, 2014; Gottlieb and Ben-Yedidia, 2014). Although such trials have been small, they are promising, inducing cross protective immunity without severe side effects.

We address here the design of epitope ensemble vaccines that have wide coverage of the human population in terms of HLA binding and wide coverage of different viral subtypes. Such an approach draws strong support from both theoretical studies (Schubert *et al.*, 2013) and from a wealth of recent experimentally verified vaccines developed with concordant goals, including *inter alia* filoviruses (Fenimore *et al.*, 2012), HIV (Ondondo *et al.*, 2016), Dengue virus (Nascimento *et al.*, 2013), Survivin (Hoffmann *et al.*, 2015) and Metapneumovirus (Li *et al.*, 2015).

In what follows, we expand on an emerging paradigm within rational vaccine design (Molero-Abraham *et al.*, 2013) to include class II epitopes, and identify multiple, conserved influenza A T cell epitopes that together comprise putative non-seasonal flu vaccines.

## 2 Methods

### 2.1 Collection of influenza A specific epitopes

Influenza A CD8+ and CD4+ T cell epitopes known to induce immune responses during natural infection were initially collected from the Immune Epitope Database and Analysis Resource [IEDB] (Peters *et al.*, 2005). Inclusion criteria restricted the data to peptides giving positive T-cell assay results and restricted by HLA molecules. In addition, for CD8+ epitopes we only considered those with 9 residues because most known epitopes processed by class I HLA are 9-mers (Reche *et al.*, 2004).

### 2.2 Collection, clustering and multiple sequence alignment of influenza A polyproteins

To generate a multiple sequence alignment (MSA), full length influenza A polyproteins were collected from the Influenza Viral Resource at the National Centre for Biotechnology Information [NCBI] (Bao *et al.*, 2008) and Influenza Research Database (Squires *et al.*, 2012). Identical sequences were removed to prevent bias when identifying conserved regions. Incomplete sequences were also omitted. Sequence sets corresponding to 12 different influenza A specific proteins were processed: each was submitted to two clustering web servers to remove redundant sequences: BLASTclust (http://toolkit.tuebingen.mpg.de/blastclust) (Altschul *et al.*, 1990) and CD-HIT (http://weizhong-lab.ucsd.edu/cdhit_suite/cgi-bin/index.cgi?cmd=cd-hit) (Fu *et al.*, 2012). To reduce sequence bias, the two clustering servers were used separately in two repeated cycles. Sequences for each influenza A protein type were clustered with 96–99% sequence similarity. Once clustered, MSA outputs were created using the multiple sequence alignment package MUSCLE (Edgar, 2004).

### 2.3 Variability analysis of influenza A polyproteins

For each influenza protein, MSA data were subjected to sequence variability analysis using the Protein Variability Server [PVS] (Garcia-Boronat *et al.*, 2008). We selected the Shannon entropy (H) as the variability metric (Stewart *et al.*, 1997) and a variability threshold of 0.5. Shannon entropy values range from 4.3 (all amino acids equally represented at a site) to 0.0 (invariant site with only one allowed residue). As a result, we obtained consensus sequences with variable positions, H > 0, masked. Subsequently, we selected as conserved epitopes those that matched precisely over their entire length with the generated consensus sequences. Thereby, we retained only those epitopes in which no amino-acid position had H > 0.5.

### 2.4 Calculation of population protection coverage

The PPC of T cell epitope ensembles equals the cumulative phenotypic frequency of the HLA alleles restricting the T cell epitopes and can be computed using the genetic frequencies of the relevant HLA alleles within the population. In this study, the PPC of HLA I-restricted epitopes was calculated using EPISOPT v.1 (http://imed.med.ucm.es/EPISOPT.html) and the IEDB PPC prediction tool (http://tools.immuneepitope.org/tools/population/iedb_input). EPISOPT computes PPC in one step after predicting epitope-HLA I binding profiles using HLA I frequencies for the five main ethnic groups present in the USA population (Black, Caucasian, Hispanic, Asian and native North American) (Cao *et al.*, 2001). The IEDB tool requires manual entry of HLA I binding profiles which we first obtained using the IEDB class I HLA binding prediction server (http://tools.immuneepitope.org/mhci/). A class I HLA reference set was used as these alleles were frequently found in the population (Weiskopf *et al.*, 2013). This allowed a PPC to be calculated for class I HLA data of the world's population rather than just the five USA ethnicities. Class I HLA binding profiles included all those alleles that were predicted to bind the peptide for the top 1% percentile rank for all peptides in the relevant antigen.

For each CD4+ T epitopes, we predicted HLA II binding affinities to different alleles found in the human population (Greenbaum *et al.*, 2011) using IEDB (http://tools.immuneepitope.org/mhcii/). Many other prediction methods are available (Soria-Guerra *et al.*, 2015). We used a 10% percentile rank cutoff to select the targeted alleles. These were then submitted to the IEDB PPC tool, allowing calculation of PPC for the world population rather than specific ethnicities.

## 3 Results

We collected from the IEDB 210 CD8+ and 816 CD4+ T cell influenza A-specific epitopes, of length 8–18 residues, known to be targeted during natural infection. We also collected influenza A proteins and clustered them using BLASTclust and CD-HIT (details in Material and Methods), obtaining two different cluster sets, and two different sets of conserved regions, one from BLASTclust and the other from CD-HIT. We used these conserved regions to identify matching conserved T cell epitopes.

First, we examined CD8+ T cell epitopes, identifying 11 conserved epitopes common to both sets of conserved regions. Additionally, two extra epitopes were identified in the BLASTclust set (GILGFVFTL, IRHENRMVL) and another two from CD-HIT (RGINDRNFW, YINTALLNA). EPISOPT was used to select CD8+ T cell epitopes for our USA-specific vaccine. Table 1 lists calculated non-zero PPC values for CD8+ epitopes generated by the server.

**Table 1.** PPC values for class I epitopes

Epitope	Profile	PPC (%)	Antigen
**FMYSDFHFI**	A0201 A0202 A0203 A0204 A0205 A0206 A0214 A2402 B4402 B5301 B5401	56.41	PA
**GILGFVFTL**	A0201 A0202 A0203 A0204 A0205 A0206 A0207 A0209 A0214 C0304	40.74	M1
**SVKEKDMTK**	A0301 A1101 A3101 A3301 A6801 B1513	32.18	PA
**ILRGSVAHK**	A0203 A0301 A1101 A3301 A6801	28.16	NP
**FYIQMCTEL**	A2402 B1509 C0702	22.05	NP
**ILKGKFQTA**	A0202 A0203 A0206 A0209 B0801	14.81	NP
**GMDPRMCSL**	A0203 A2402 B0801	14.18	NP
**YYLEKANKI**	A0214 B3801 B39011	3.92	PA
**RGINDRNFW**	B1513 B5701 B5702	2.48	NP
**TQIQTRRSF**	B5701	1.93	PB1
**DTVNRTHQY**	A6601 B5702 B5801	1.53	PB1

9-mer CD8+ epitopes able to induce a T cell response against influenza A. Each epitope's individual PPC and HLA class II allelic binding restriction is also given. Only epitopes with non-negligible PPCs are shown.

To reach a PPC > 80% a minimum of two epitopes were needed. To achieve a PPC > 95% at least six epitopes are required. EPISOPT identified four different combinations of which two combinations reached a PPC of 95.7% and the other two 95.4%. We selected set one to define the CD8+ arm of our USA-specific ensemble vaccine.

We collected 816 influenza A CD4+ T cell epitopes from the IEDB database and selected 7 conserved CD4+ T cell epitopes by comparing with the conserved regions identified by BLASTClust and CD-HIT clustering routes were used. These conserved epitopes of varying amino acid length were submitted to IEDB the class II HLA binding prediction tool (Table 2). Many epitopes did not bind to any alleles within the top 10% percentile rank and of those that did only one epitope, TGTGYTMDTVNRTHQ, was identified by both BLASTclust and CD-HIT. Three additional epitopes were identified by BLASTclust (KGILGFVFTLTVPSE, ILGFVFTLTVPSERG and NPLIRHENRMVLAST) and three by CD-HIT (TYQRTRALVRTGMDP, MAFLEESHPGIFENS and EYIMKGVYINTALLN).

**Table 2.** PPC values for class II epitopes

Epitope	Profile	PPC (%)	Antigen
**KGILGFVFT**	DPA1*01/DPB1*04:01 DRB1*11:01	66.92	M1
** LTVPSE**	DPA1*03:01/DPB1*04:02 DRB1*04:05 DPA1*02:01/DPB1*14:01 DRB1*04:01 DPA1*01:03/DPB1*02:01 DRB1*08:02 DQA1*04:01/DQB1*04:02 DRB5*01:01 DPA1*02:01/DPB1*05:01 DRB1*07:01 DRB1*15:01 DRB1*03:01		
**EYIMKGVYI**	DQA1*01:02/DQB1*06:02 DRB1*08:02	62.27	PA
** NTALLN**	DPA1*02:01/DPB1*01:01 DRB3*01:01 DPA1*03:01/DPB1*04:02 DRB1*11:01 DPA1*01:03/DPB1*02:01 DRB1*04:05 DRB5*01:01 DRB1*04:01 DRB1*15:01 DRB1*03:01 DRB1*12:01 DRB1*13:02		
**NPLIRHENR**	DRB1*03:01 DRB3*02:02 DRB1*08:02	61.62	M1
** MVLAST**	DRB1*15:01 DQA1*01:02/DQB1*06:02 DRB1*09:01 DRB1*04:01 DRB1*13:02 DRB3*01:01 DRB1*11:01		
**ILGFVFTLT**	DPA1*01/DPB1*04:01 DRB1*11:01	59.12	M1
** VPSERG**	DPA1*03:01/DPB1*04:02 DRB1*04:05 DPA1*02:01/DPB1*14:01 DRB1*04:01DQA1*04:01/DQB1*04:02 DRB5*01:01 DRB1*07:01 DRB1*08:02 DQA1*05:01/DQB1*02:01 DRB1*03:01 DRB3*02:02 DRB1*09:01 DPA1*01:03/DPB1*02:01		
**TGTGYTMD**	DRB3*01:01 DRB1*03:01 DRB1*04:01	27.97	PB1
**TVNRTHQ**	DRB3*02:02		
**TYQRTRAL**	DRB5*01:01 DRB1*07:01 DRB1*11:01	27.73	NP
**VRTGMDP**	DRB3*02:02		
**MAFLEESHPGIFENS**	DRB3*01:01 DRB1*13:02 DRB1*09:01	12.87	PB1

Summary of selected CD4+ epitopes, with their predicted class II binding restrictions and PPC.

All epitope-HLA II binding profiles were then entered into the IEDB PPC tool for analysis. NPLIRHENRMVLAST, TGTGYTMDTVNRTHQ, TYQRTRALVRTGMDP and MAFLEESHPGIFENS have class II HLA binding profiles that are completely covered by the other three remaining epitopes and therefore they were not considered when calculating PPC with multiple CD4+ T cell epitopes. The remaining three CD4+ T cell epitopes gave a combined PPC of 76.4%. The resulting combination of selected CD8+ and CD4+ epitopes form a population-weighted potential candidate USA-specific multiple epitope ensemble vaccine capable of inducing region-specific cross protection against influenza A (Table 3).

**Table 3.** Components of USA-specific vaccine

Epitope	Profile	PPC (%)	Antigen
**GILGFVFTL**	A0201 A0202 A0203 A0204 A0205 A0206 A0207 A0209 A0214 C0304	40.7	M1
**FMYSDFHFI**	A0201 A0202 A0203 A0204 A0205 A0206 A0214 A2402 B4402 B5301 B5401	56.4	PA
**GMDPRMCSL**	A0203 A2402 B0801	14.2	NP
**SVKEKDMTK**	A0301 A1101 A3101 A3301 A6801 B1513	32.2	PA
**FYIQMCTEL**	A2402 B1509 C0702	22.2	NP
**DTVNRTHQY**	A6601 B5702 B5801	1.5	PB1
**KGILGFVFTL**	DPA1*01/DPB1*04:01 DRB1*11:01	66.9	M1
**TVPSE**	DPA1*03:01/DPB1*04:02 DRB1*04:05 DPA1*02:01/DPB1*14:01 DRB1*04:01 DPA1*01:03/DPB1*02:01 DRB1*08:02 DQA1*04:01/DQB1*04:02 DRB5*01:01 DPA1*02:01/DPB1*05:01 DRB1*07:01 DRB1*15:01 DRB1*03:01		
**EYIMKGVYIN**	DQA1*01:02/DQB1*06:02	62.3	PA
**TALLN**	DRB1*08:02 DPA1*02:01/DPB1*01:01 DRB3*01:01 DPA1*03:01/DPB1*04:02 DRB1*11:01 DPA1*01:03/DPB1*02:01 DRB1*04:05 DRB5*01:01 DRB1*04:01 DRB1*15:01 DRB1*03:01 DRB1*12:01		
**ILGFVFTLTV**	DRB1*13:02 DPA1*01/DPB1*04:01	59.1	M1
**PSERG**	DRB1*11:01 DPA1*03:01/DPB1*04:02 DRB1*04:05 DPA1*02:01/DPB1*14:01 DRB1*04:01 DQA1*04:01/DQB1*04:02 DRB5*01:01 DRB1*07:01 DRB1*08:02 DQA1*05:01/DQB1*02:01 DRB1*03:01 DRB3*02:02 DRB1*09:01 DPA1*01:03/DPB1*02:01		

CD8+ and CD4+ epitopes comprising our USA-specific vaccine, together with origin, individual PPC and HLA class II allelic binding restriction.

Table 3 lists the CD8+ and CD4+ epitopes comprising our USA-specific vaccine, together with origin, individual PPC and HLA class II allelic binding restriction.

To derive the equivalent universal vaccine, the class I HLA binding profiles of each epitope were predicted using the IEDB class I HLA binding prediction tool. We then used the IEDB PPC tool to estimate PPC values against the world population, rather than the North American-only coverage provided by EPISOPT. GMDPRMCSL, YINTALLNA and IRHENRMVL were excluded as they did not bind to any class I HLA allele within the top 1% percentile rank. Excluding those epitopes that gave a PPC of 0%, a maximum PPC of 91.04% was possible with the remaining 12 CD8+ epitopes. This was different result to EPISOPT v.1, where just 6 epitopes gave a PPC > 95%. This reflects the lesser diversity within the North America population compared to the world population as a whole. Epitopes which gave an individual PPC > 10% using the IEDB PPC tool were considered; and together reached a PPC value of 90.2%. These constituted the CD8+ component of our universal influenza vaccine (Table 4). The selected CD4+ and CD8+ epitopes were combined together to calculate a PPC against both class I HLA and class II HLA alleles against the world's population using the IEDB PPC tool (Table 4). A total PPC of 97.7% was achieved. The selected T cell epitopes form a population-weighted potential multiple epitope ensemble vaccine able to induce broad, global, cross-protection against influenza A.

**Table 4.** Components of universal vaccine

Epitope	Profile	PPC (%)	Antigen
**GILGFVFTL**	A0201 A0202 A0203 A0204 A0205 A0206 A0207 A0209 A0214 C0304	42.7	M1
**FMYSDFHFI**	A0201 A0202 A0203 A0204 A0205 A0206 A0214 A2402 B4402 B5301 B5401	49.5	PA
**ILRGSVAHK**	A0203 A0301 A1101 A3301 A6801	20.4	NP
**FYIQMCTEL**	A2402 B1509 C0702	26.2	NP
**ILKGKFQTA**	A0202 A0203 A0206 A0209 B0801	10.6	NP
**YYLEKANKI**	A0214 B3801 B39011	26.2	PA
**VSDGGPNLY**	A0101 A3002	19.6	PB1
**YSHGTGTGY**	A0101 A2601 B1502	29.2	PB1
**KGILGFVFTL**	DPA1*01/DPB1*04:01 DRB1*11:01	66.9	M1
**TVPSE**	DPA1*03:01/DPB1*04:02 DRB1*04:05 DPA1*02:01/DPB1*14:01 DRB1*04:01 DPA1*01:03/DPB1*02:01 DRB1*08:02 DQA1*04:01/DQB1*04:02 DRB5*01:01 DPA1*02:01/DPB1*05:01 DRB1*07:01 DRB1*15:01 DRB1*03:01		
**EYIMKGVYIN**	DQA1*01:02/DQB1*06:02 DRB1*08:02	62.3	PA
**TALLN**	DPA1*02:01/DPB1*01:01 DRB3*01:01 DPA1*03:01/DPB1*04:02 DRB1*11:01 DPA1*01:03/DPB1*02:01 DRB1*04:05 DRB5*01:01 DRB1*04:01 DRB1*15:01 DRB1*03:01 DRB1*12:01 DRB1*13:02		
**ILGFVFTLTV**	DPA1*01/DPB1*04:01 DRB1*11:01	59.1	M1
**PSERG**	DPA1*03:01/DPB1*04:02 DRB1*04:05 DPA1*02:01/DPB1*14:01 DRB1*04:01 DQA1*04:01/DQB1*04:02 DRB5*01:01 DRB1*07:01 DRB1*08:02 DQA1*05:01/DQB1*02:01 DRB1*03:01 DRB3*02:02 DRB1*09:01 DPA1*01:03/DPB1*02:01		

CD8+ and CD4+ epitopes comprising our global vaccine, together with origin, individual PPC and HLA class II allelic binding restriction.

Table 4 lists the CD8+ and CD4+ epitopes comprising our global vaccine, together with origin, individual PPC and HLA class II allelic binding restriction.

We have intentionally not specifically targeted subtypes. However, due to their wide sequence conservation, the identified epitopes are well represented across most major subtypes. Apart from subtypes of influenza A unique to bats, there are 16 haemagglutinin and 9 neuraminidase subtypes transmissible to humans making 144 possible H*N* combinations, of which 128 have been observed in the wild, as indicated by the presence of at least one designated sequence in the Influenza Virus Resource (Bao *et al.*, 2008), as of February 2016. BLASTing each of our 9 USA-specific vaccine epitopes against Orthomyxoviridae (taxid:11308) sequences within the non-redundant NCBI protein database, generated sets of 100% identical matches to sequences from distinct H*N* subtypes, ranging in number from 95 H*N* subtypes (GILGFVFTL) to 111 subtypes (SVKEKDMTK). As a whole, the USA-specific vaccine has sequence matches to a total of 117 distinct H*N* sub-types. This indicates that the vaccine has a high coverage (95%) of observed influenza subtypes. BLASTing the 11 universal vaccine epitopes against Orthomyxoviridae sequences, produced 100% identical matches to distinct H*N* subtypes, which ranged in number from 93 (KGILGFVFTLTVPSE) to 108 (FMYSDFHFI). The universal vaccine matches 113 H*N* sub-types, again indicating a high overall coverage (88%) of observed subtypes. Identifying additional highly subtype-specific conservation might enhance our putative universal Influenza A vaccine further, as adding a cocktail of strain-specific epitopes may strengthen cross-protection within our putative epitope ensemble.

No single epitope vaccine would not provide universal protection against heterosubtypic influenza A strains. No single peptide vaccines, reinforcing the need to examine multiple conserved epitopes. Individually, no single peptide gave a PPC greater >90% however, such values were achieved when combining epitopes. It is evident from a comparison of EPISOPT and IEDB that generated PPC values are method-dependent, with alternative allele frequencies and calculation strategies yielding different values. Nonetheless, high consensus values, as derived here, indicate the value of this approach as a fully validated starting point for vaccine design. Ideally, epitopes with a broad allele specificity would be assayed experimentally against well-understood alleles representative of class I and class II supertypes (Doytchinova and Flower, 2005; Doytchinova *et al.*, 2005).

All T cell epitopes included in the final vaccine combination were either part of polymerase acidic protein (PA), nucleoprotein (NP), matrix 1 protein (M1) or polymerase basic protein 1 (PB1). The large PPC value found for GILGFVFTL located on M1 protein is not surprising as previous research concluded it was broadly recognized by the population (Alexander *et al.*, 2010a). It is known to be immunodominant when restricted by HLA-A02 and other evidence suggests degenerate recognition by CTL when restricted by HLA-C08 (Choo *et al.*, 2014). In cellular immunity, immunodominance refers to the observation that while many peptides can be presented by host cells, a larger portion of T cells focus their attention on a very limited number of HLA-presented peptides. Such evidence, together with our results, supports the view that the epitope will be useful in providing broad protection against distinct influenza A strains.

NP has long been considered a viable option for inducing cross protective CTL in humans and mice; many studies have sought to ensure NP-based vaccines are immunogenic enough to be cross protective. These include priming with DNA vaccines containing NP (Epstein *et al.*, 2005) alone or combined with other conserved proteins such as NS1 and M1 (Zhirnov *et al.*, 2007) followed by boosting with recombinant NP derived from *E.coli*. In animal models, all show promise, with cross-reactive T cell responses against influenza A subtypes observed at many levels. A vaccine consisting of modified vaccinia virus Ankara vector, M1 and NP (MVA-NP + M1) has also been tested in clinical trials involving healthy adults (Huang *et al.*, 2012). The MVA-NP + M1 vaccine yields much higher T cell responses compared to other influenza vaccines, as well as being safe in animal models and human trials. T cell responses were boosted when combined with seasonal vaccination compared to seasonal vaccination alone suggesting seasonal vaccines may still prove a useful component in future universal vaccines (Antrobus *et al.*, 2014a,b; Berthoud *et al.*, 2011).

Alexander *et al.* (2010b) also identified FMYSDFHFI, FYIQMCTEL, YYLEKANKI, GILGFVFTL and YSHGTGTGY as CD8+ epitopes for use in cross protective vaccines. Similar to our results, each binds extensively within a particular class I HLA supertype: FMYSDFHFI, A2; FYIQMCTEL, A24; YYLEKANKI, A24; GILGFVFTL, A2 and YSHGTGTGY, A1. These epitopes were conserved in over 93% of 69 distinct influenza strains and 100% conserved in 10 swine flu H1N1 strains. All these epitopes produce good CTL responses in donors expressing the relevant class I HLA alleles. The similarity of derived epitopes coupled to their high conservation among a diverse group of influenza A strains, suggests such epitopes will prove viable targets for future influenza A epitope vaccine formulations.

None of these highly conserved CD8+ or CD4+ T cell epitopes were found in hemagglutinin (H) or neuraminidase (N). While it is often assumed wrongly that H or N epitopes are all important, this is not the case with T-cell responses. While N and H are the key proteins for antibody binding, cellular immunity can respond to viral infection before viral shedding, opening the whole influenza genome to immune surveillance. The absence of N and H epitopes is explained by the low sequence conservation between different strains (Chen and Deng, 2009), due to the highly variable antigen binding sites that allow antibody immune evasion.

Recent studies suggest immunodominant influenza epitopes are less conserved generally than their subdominant counterparts, which is thought to result from immune pressure (Chen *et al.*, 2014). Immunodominant epitopes are likely to give the greatest immune response. Some of our epitopes may be subdominant potentially reducing their ability to induce potent immune responses. However, immunodominance hierarchies are dynamic and can be altered by vaccination (Welsh *et al.*, 2010). Any peptide that is efficiently processed and presented in both target and antigen-presenting cells are good candidates for vaccines regardless of their immunodominance. Only subdominance resulting from failures of processing or from poor binding to MHCs would lead to an ineffective vaccine. However, the epitopes we selected are likely to be processed in vivo as they are known to be targeted during a natural infection and exhibit high predicted MHC binding.

While the performance of many class I binding algorithms has recent improved significantly, class II predictions remain poor or inconsistent (Lafuente and Reche, 2009). Since class II HLA have open-ended binding sites, they can bind peptides with a much broader length distribution than class I. The lower quality performance compared to class I HLA prediction increases the likelihood of identifying false-positive CD4+ T cell epitopes (Nielsen *et al.*, 2010). In this study, CD8+ epitopes were restricted to 9-mer epitopes, even though class I HLA molecules can present a significant number of peptides of other lengths (MacDonald *et al.*, 2010). Although most known class I HLA specific peptides are 9-mers, and class I HLA binding predictions of peptides of other lengths are not as reliable (Lundegaard *et al.*, 2010), many immunogenic CD8+ epitopes of length other than 9 are known: for example, Alexander *et al.* (2010) identified multiple 10-mer, influenza A specific CD8+ epitopes. Future studies might therefore usefully include epitopes of different lengths.

The list of epitopes identified here does not, in itself, constitute the entirety of a deployable vaccine. Constructing a multiple epitope ensemble vaccine is often problematic as they are seldom sufficiently immunogenic. Short peptides suffer from poor immunogenicity and therefore vaccines require additional components to help initiate T cell responses. Epitopes must be delivered, packaged into a stable vaccine formulation (Yang and Kim, 2015). Epitopes can be delivered as poly-epitope peptide(s) or as part of a viral vector. In either case, the order of epitopes and the presence of cleavage sites, etc. is crucial. Schubert and Kohlbacher (2016) have, for example, addressed how to optimize this process. The typical lack of immunogenicity of such constructs is notable (Mahanty et al., 2015), and much empirical work—including optimizing the number and timings of vaccination—is necessary to address this (De Groot et al., 2005a,b). The main way to do this is by addition of adjuvants to the formulation (Bayry et al., 2008).

Our approach has focused on designing the so-called biological component of a vaccine, the part responsible—through molecular recognition events—for engendering the specificity of vaccine responses. Issues will need to be addressed, including the synergistic orchestration of both the mechanistic targeting and/or co-uptake of vaccine components and the optimal logistics of vaccination protocols, such as chosen regimen and the size, number and frequency of vaccinations (Moyer *et al.*, 2016). In this regard, computational modelling frameworks have much to offer, allowing us to predict overall immune system response dynamics and pharmacokinetics that should allow us to optimize memory effects and the dosing of vaccine administration (Pappalardo *et al.*, 2014).

## 4 Conclusion

A ‘universal’ vaccine would ideally induce immunity against all or most influenza A subtypes. However, as influenza continually evolves, formulating a vaccine with such broad protection remains problematic. Current seasonal influenza vaccines lack the ability to induce cellular immunity and instead induce strain specific humoral immunity where novel or differing influenza strains can easily bypass immune recognition. In this study, multiple, conserved T cell epitopes were identified using immunoinformatics. The combination of epitopes identified here should be able to induce broad heterosubtypic, protection against influenza A across the global population (PPC= 97.7%) or in geographically restricted populations, in this case the USA (PPC = 96.3%). Our putative vaccines contain both CD8+ and CD4+ epitopes, as both types of T cells play important roles in viral clearance. Although all identified epitopes are known to be immunoreactive, further studies are needed to assess their efficacy. The extent of cross protection will require further analysis to verify the efficacy against particular influenza A subtypes. Despite these limitations the results are a promising vindication of our approach, and the extensions we detail, should prove of interest in future influenza vaccination strategies.
